# Synergistic cell-cycle repression by HDAC/EZH2 co-targeting in H3K27-altered diffuse midline glioma

**DOI:** 10.1016/j.omton.2026.201306

**Published:** 2026-07-24

**Authors:** Zili Zhen, Qiang Gao, Yong Ai, Zhihong Qian, Yukui Shang, Yafang Deng, Shuai Chen, Xin Ni, Wei Zhang

**Affiliations:** 1 IDG/McGovern Institute for Brain Research, School of Life Sciences, Tsinghua University, Beijing 100084, China; 2 Department of Neurosurgery, General Hospital of Ningxia Medical University, Yinchuan, Ningxia 750003, China; 3 School of Clinical Medicine, Tsinghua Medicine, Tsinghua University, Beijing 100084, China; 4 Department of Neurosurgery, Beijing Children’s Hospital, Capital Medical University, National Center for Children’s Health, Beijing 100045, China; 5 The State Key Laboratory for Complex, Severe, and Rare Diseases, School of Basic Medical Sciences, Tsinghua Medicine, Tsinghua University, Beijing 100084, China; 6 Department of Internal Medicine, Peking Union Medical College Hospital, Beijing 100730, China; 7 Neurological Center, Beijing Children’s Hospital Shunyi Women’s and Children’s Hospital, Beijing 101300, China; 8 National Center for Pediatric Cancer Surveillance, Beijing Children’s Hospital, Capital Medical University, National Center for Children’s Health, Beijing 100045, China; 9 Clinical Research Center, Beijing Children’s Hospital, Capital Medical University, National Center for Children’s Health, Beijing 100045, China

**Keywords:** diffuse midline glioma, epigenetic therapy, HDAC inhibitor, EZH2 inhibitor, cell-cycle arrest, glioma stem cell, panobinostat, tazemetostat, therapeutic synergy

## Abstract

Diffuse midline glioma (DMG) is an aggressive malignancy driven by the H3K27M oncohistone, leading to global epigenetic dysregulation and limited efficacy of single-agent epigenetic therapies. We previously observed clinical benefit from an HDAC/EZH2 inhibitor-based therapeutic regimen, but the mechanistic basis was unclear. A panel of patient-derived DMG glioma stem cell (GSC) cultures from brainstem and spinal cord tumors was used for drug-response and functional assays, with representative models further analyzed for transcriptomic alterations, cell-cycle distribution, global histone-modification changes, self-renewal, and *in vivo* efficacy. Antitumor efficacy was validated in orthotopic xenografts. Panobinostat and tazemetostat showed robust synergy across patient-derived DMG GSC cultures, suppressing GSC growth, proliferation, and self-renewal while inducing apoptosis and G0/G1 cell-cycle arrest. Transcriptomic analyses revealed coordinated repression of cell-cycle programs and downregulation of OPC-like and stemness-associated programs, accompanied by the induction of differentiation-associated neuronal genes. Functionally, dual treatment reduced stem-cell frequency by >90% to >99%. In orthotopic xenografts, combination therapy significantly decreased tumor burden, reduced spinal dissemination, and prolonged survival. Together, these findings indicate that dual HDAC/EZH2 inhibition disrupts epigenetically maintained proliferative and stem-like programs through coordinated cell-cycle repression, apoptosis induction, and impaired self-renewal. This work supports HDAC/EZH2 co-targeting as a rational therapeutic backbone for H3K27-altered DMG and its incorporation into future rational combination strategies.

## Graphical abstract

**Figure:**
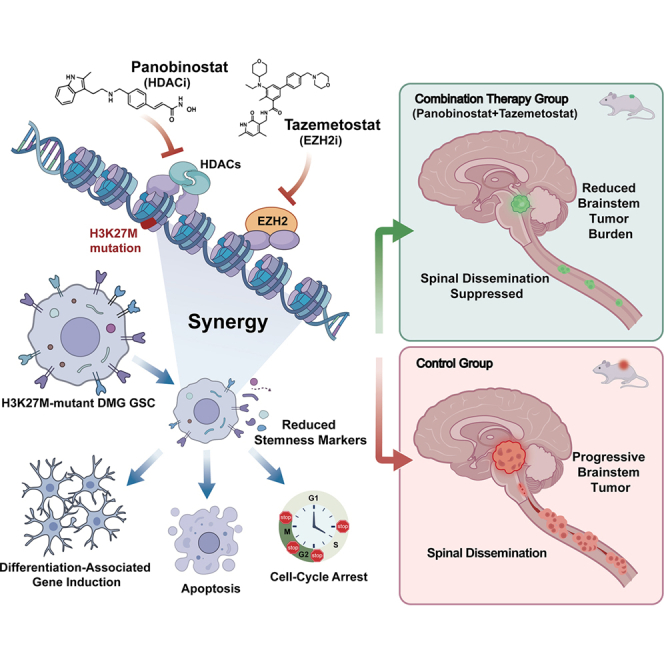

## Teaser

Diffuse midline glioma (DMG) is a lethal H3K27-altered tumor lacking effective therapies. Dual panobinostat/tazemetostat treatment synergistically represses cell-cycle programs, induces apoptosis, suppresses DMG stem-like programs, impairs self-renewal capacity, and prolongs survival in orthotopic models, providing a mechanistic and translational rationale for clinical evaluation of HDAC/EZH2 co-targeting in DMG.

## Introduction

Diffuse midline glioma (DMG), H3K27-altered, is a highly aggressive malignancy arising in critical midline structures, such as the thalamus, brainstem, and spinal cord.^1^^,^^2^ Its diffuse infiltrative growth pattern renders gross total resection unachievable, and most patients undergo stereotactic or robot-assisted biopsy solely for molecular diagnosis.^3^ The resulting scarcity of tumor tissue has limited biological characterization and hindered therapeutic development. To date, no consensus treatment guidelines have been established by the National Comprehensive Cancer Network (NCCN), and current radiotherapy-based regimens yield a median overall survival of merely 11 months and a dismal 2-year survival rate of <10%, outcomes even poorer than glioblastoma, another WHO grade 4 malignancy.^4^

A defining molecular event in DMG is the H3K27M mutation, in which lysine-to-methionine substitution at histone H3 position 27 disrupts polycomb repressive complex 2 (PRC2) function.^5^ This dominant-negative effect leads to global loss of H3K27 trimethylation (H3K27me3) and aberrant accumulation of H3K27 acetylation (H3K27ac),^6^^,^^7^^,^^8^ reshaping chromatin architecture and driving oncogenic transcriptional programs. Enhancer of zeste homolog 2 (EZH2) is the catalytic subunit of the PRC2 complex, which silences gene expression through H3K27me3 deposition, whereas histone deacetylase (HDAC) activity modulates H3K27ac levels. Epigenetic perturbation using tazemetostat (an EZH2 inhibitor) can derepress tumor suppressors such as *CDKN2A*, while panobinostat (a pan-HDAC inhibitor) broadly remodels chromatin accessibility and transcriptional programs through modulation of histone acetylation.^9^^,^^10^^,^^11^^,^^12^^,^^13^^,^^14^

This bidirectional disruption creates a distinctive therapeutic paradox: although H3K27M tumors exhibit elevated H3K27ac and reduced H3K27me3, inhibiting both HDAC/EZH2 has shown unexpected antitumor activity. In our prior compassionate-use clinical series, combined panobinostat and tazemetostat-based immunotherapy elicited prolonged survival in patients with recurrent DMG,^15^ including an individual with extensive leptomeningeal metastasis who survived 20 months with improved quality of life, remarkably longer than 1–3 months of overall survival in historical cohorts,^16^ while three additional recurrent or progressive cases survived 33, 27, and 16 months, respectively.^15^ These observations suggest that coordinated modulation of opposing epigenetic regulators may uncover latent vulnerabilities not accessible to single-agent therapy. However, the biological mechanisms underlying this synergy remain undefined.

**Figure 1:**
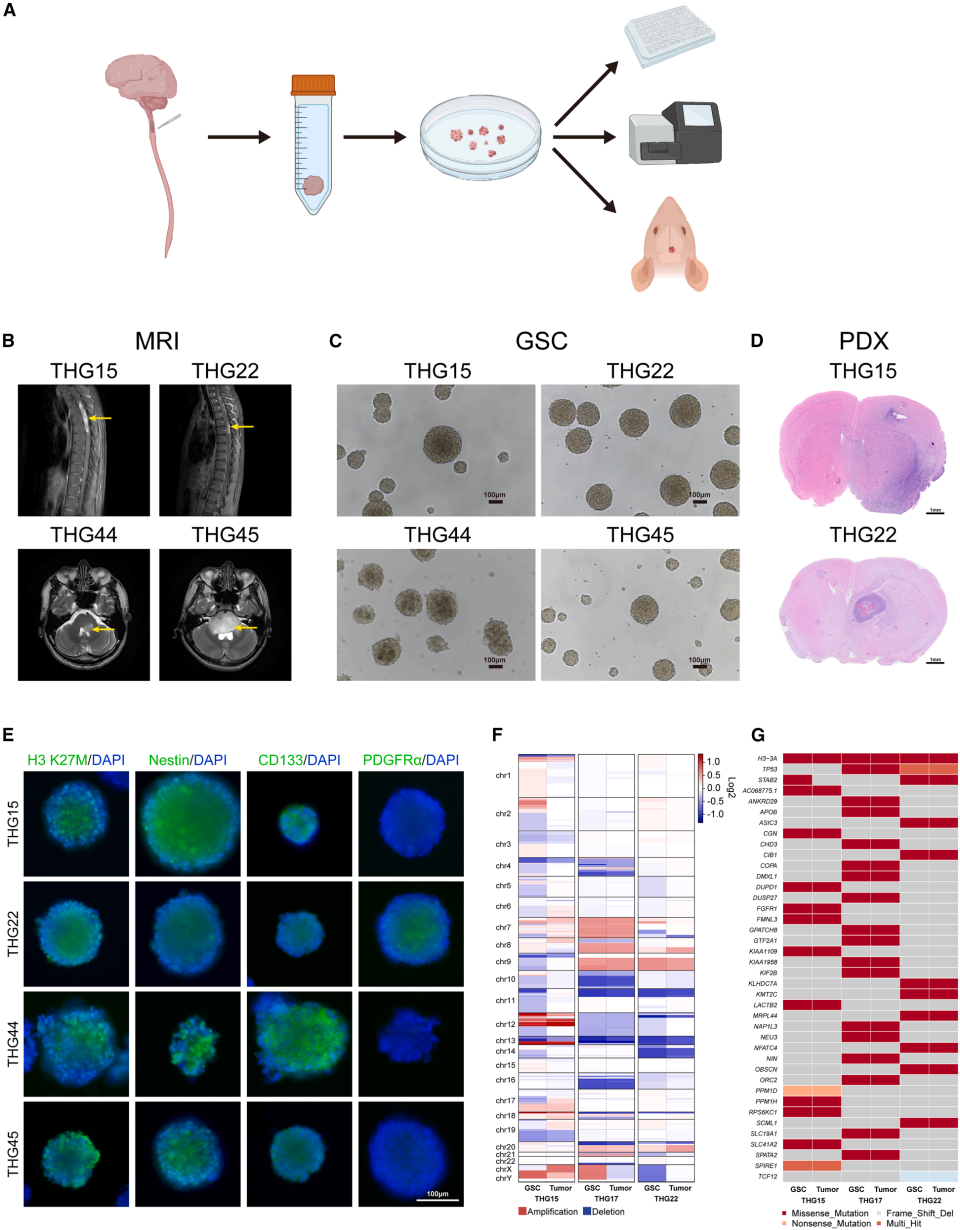
Establishment and characterization of patient-derived *in vitro* and *in vivo* models of DMG (A) Schematic illustration of the generation of patient-derived DMG GSC cultures for downstream high-throughput sequencing analyses and *in vivo* assays. (B) Magnetic resonance imaging (MRI) scans showing the primary tumors from which the patient-derived DMG GSC cultures were established. THG15 was derived from a previously reported recurrent spinal DMG case, whereas the remaining cultures were derived from treatment-naive primary tumors. Yellow arrows indicate the tumor regions. (C) Representative light microscopy images of the four GSC cultures (THG15, THG22, THG44, and THG45) showing characteristic tumor sphere structures *in vitro*. Scale bars, 100 μm. (D) Hematoxylin and eosin (H&E)-stained sections showing tumor formation following stereotactic injection of THG15 and THG22 GSCs in a murine xenograft model. Densely stained hematoxylin (blue-purple) areas indicate high tumor cellularity. Scale bars, 1 mm. (E) Representative immunofluorescence staining of DMG neurospheres for H3K27M, Nestin (neural stem cell marker), CD133 (cancer stem cell marker), and PDGFRα (oligodendrocyte progenitor cell marker). Scale bars, 100 μm. (F) Genome-wide copy number variation (CNV) profiles comparing GSC models (THG15, THG17, and THG22) with their matched parental tumor tissues. Red indicates genomic amplification and blue indicates copy number loss. (G) Mutational landscapes of key driver genes in GSC cultures (THG15, THG17, and THG22) and their matched parental tumor tissues. Mutation types are color-coded, and gray represents wild-type status.

Here, we systematically investigate the therapeutic effects and molecular mechanisms of dual HDAC/EZH2 inhibition using patient-derived H3K27-altered DMG glioma stem cell (GSC) models (Figure 1A). We demonstrate robust synergy between panobinostat and tazemetostat across diverse *in vitro* and *in vivo* systems, accompanied by suppression of proliferative and stemness-associated programs. By dissecting how simultaneous perturbation of the altered H3K27ac/H3K27me3 axis produces integrated transcriptional, functional, and global histone-modification responses, this study provides a mechanistic rationale for HDAC/EZH2 co-targeting in DMG.

## Results

### Establishment and validation of patient-derived DMG GSC models

To examine the synergistic effects of epigenetic agents, we first established five primary H3K27M-mutant DMG GSC cultures, designated THG15, THG17, THG22, THG44, and THG45. For functional drug-response studies, we focused on four cultures with sufficient proliferative capacity, including two spinal cord-derived models, THG 15 and THG22, and two brainstem-derived models, THG44 and THG45 (Figure 1B). Molecular pathology confirmed the H3.3K27M mutation in all cases (Table S1). Notably, the THG15 sample was obtained from a recurrent case post-surgery, whereas all other patient samples were treatment-naive prior to collection. Dissociated cells from each tumor efficiently generated free-floating neurospheres 100–300 μm in diameter after 7 days (Figure 1C) and formed infiltrative orthotopic xenografts in nude mice (Figure 1D). To characterize lineage markers within the neurospheres, we performed *in situ* immunofluorescence staining. All cultures exhibited nuclear expression of H3K27M protein (Figure 1E) and robust expression of the neural stem cell marker Nestin.^17^ CD133 expression was detected across the cultures.^18^^,^^19^ The oligodendrocyte progenitor cell (OPC) marker platelet-derived growth factor receptor alpha (PDGFRα) displayed heterogeneous expression, with strong positivity observed primarily in THG22.^20^^,^^21^ The orthotopic xenograft model retained H3K27M expression and showed detectable stem/progenitor-associated marker expression *in vivo* (Figure S1). Whole-exome sequencing demonstrated high genomic concordance between available matched GSC cultures, including THG15, THG17, and THG22, and their corresponding primary tumors, with preservation of concordant copy-number alterations and somatic mutations (Figures 1F and 1G).^22^^,^^23^ Collectively, these findings confirm that our patient-derived H3K27M-mutant GSC models faithfully recapitulate key biological and molecular features of DMG and provide a robust platform for evaluating therapeutic interventions.

### Panobinostat and tazemetostat synergistically inhibit DMG growth *in vitro*

**Figure 2:**
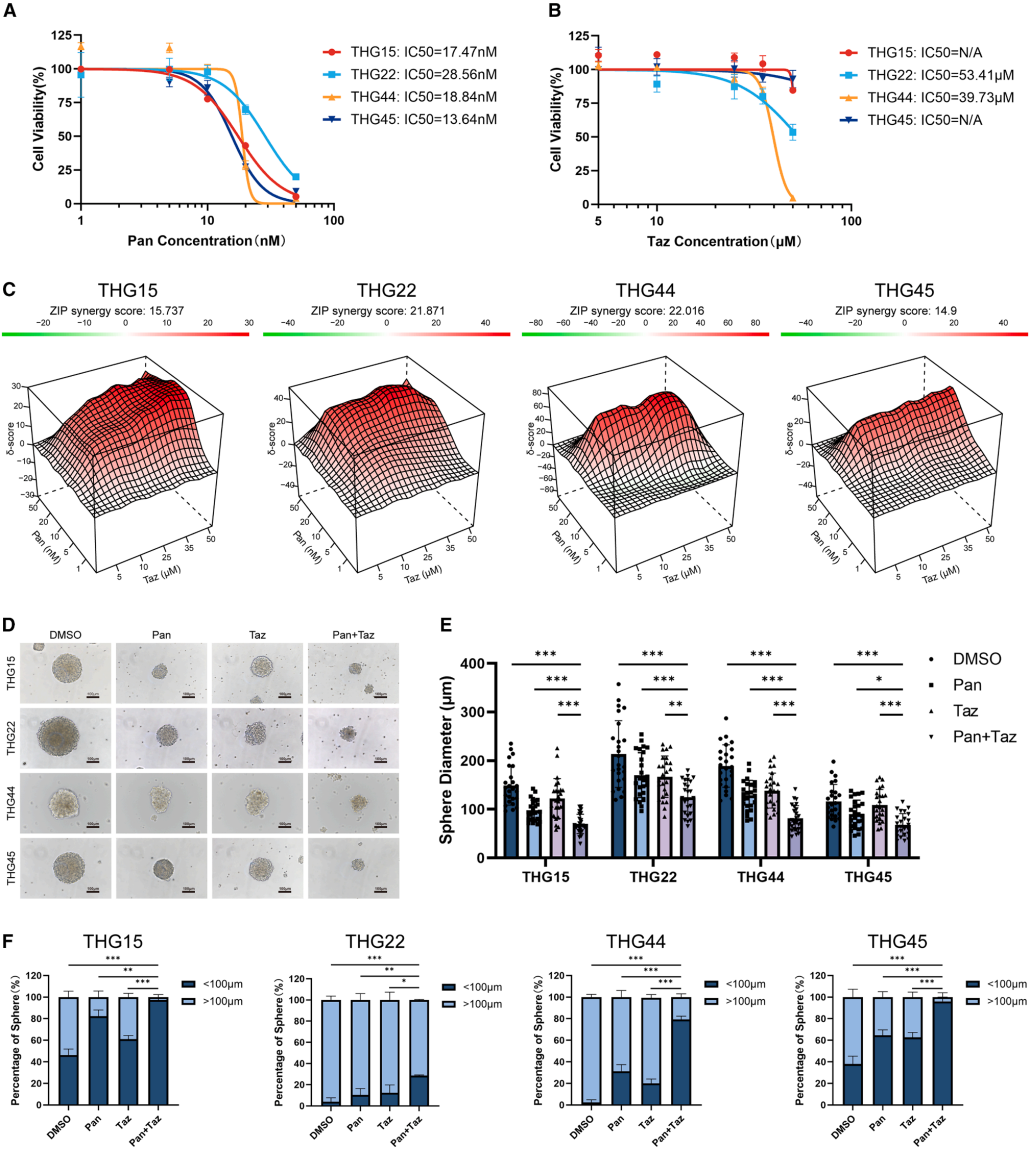
Panobinostat and tazemetostat synergistically inhibit DMG GSC growth *in vitro* (A) Dose-response curves and corresponding half-maximal inhibitory concentrations (IC_50_) for panobinostat in the four DMG GSC cultures. (B) Dose-response curves and corresponding IC_50_ values for tazemetostat in the DMG GSC cultures. (C) Three-dimensional synergy landscapes for panobinostat and tazemetostat in each GSC culture. Synergistic and antagonistic dose combinations are shown in red and green, respectively. A ZIP synergy score >10 indicates significant drug synergy. (D) Representative phase-contrast images of GSC neurospheres following the indicated treatments. Scale bars, 100 μm. (E) Quantification of neurosphere diameters following drug treatment. (F) Percentage of efficiently formed neurospheres (>100 μm in diameter) following drug treatment. Pan, panobinostat; Taz, tazemetostat; ∗*p* < 0.05; ∗∗*p* < 0.01; ∗∗∗*p* < 0.001.

To assess potential synergy between panobinostat and tazemetostat, we first evaluated dose-response relationships for each monotherapy across four patient-derived GSC cultures (Figure 2A). Panobinostat effectively inhibited growth in all cultures, with IC_50_ values of 17.47 nM (THG15), 28.56 nM (THG22), 18.84 nM (THG44), and 13.64 nM (THG45). In contrast, tazemetostat showed appreciable inhibitory activity only in THG22 and THG44, with IC_50_ values of 53.41 and 39.73 μM, whereas THG15 and THG45 remained minimally responsive within the tested concentration range (Figure 2B). Synergy was then quantified using checkerboard combination assays. Zero interaction potency (ZIP) synergy scores exceeded 10 in all four cultures (Figures 2C, S2A, and S2B), indicating robust synergistic interactions. Accordingly, the optimal synergistic concentration pairs were selected for subsequent experiments (Table S2). Furthermore, the combination treatment significantly reduced cell viability compared to the respective monotherapies (Figure S2C). To determine effects on self-renewal, we performed sphere-formation assays. Both agents reduced neurosphere size relative to controls, but the combination further suppressed neurosphere diameter and formation efficiency (Figures 2D–2F). Collectively, these findings demonstrate that panobinostat and tazemetostat synergistically inhibit growth and reduce clonogenic capacity in patient-derived DMG GSCs *in vitro*.

### Combined panobinostat and tazemetostat suppress DMG growth and prolong survival in the orthotopic model

**Figure 3:**
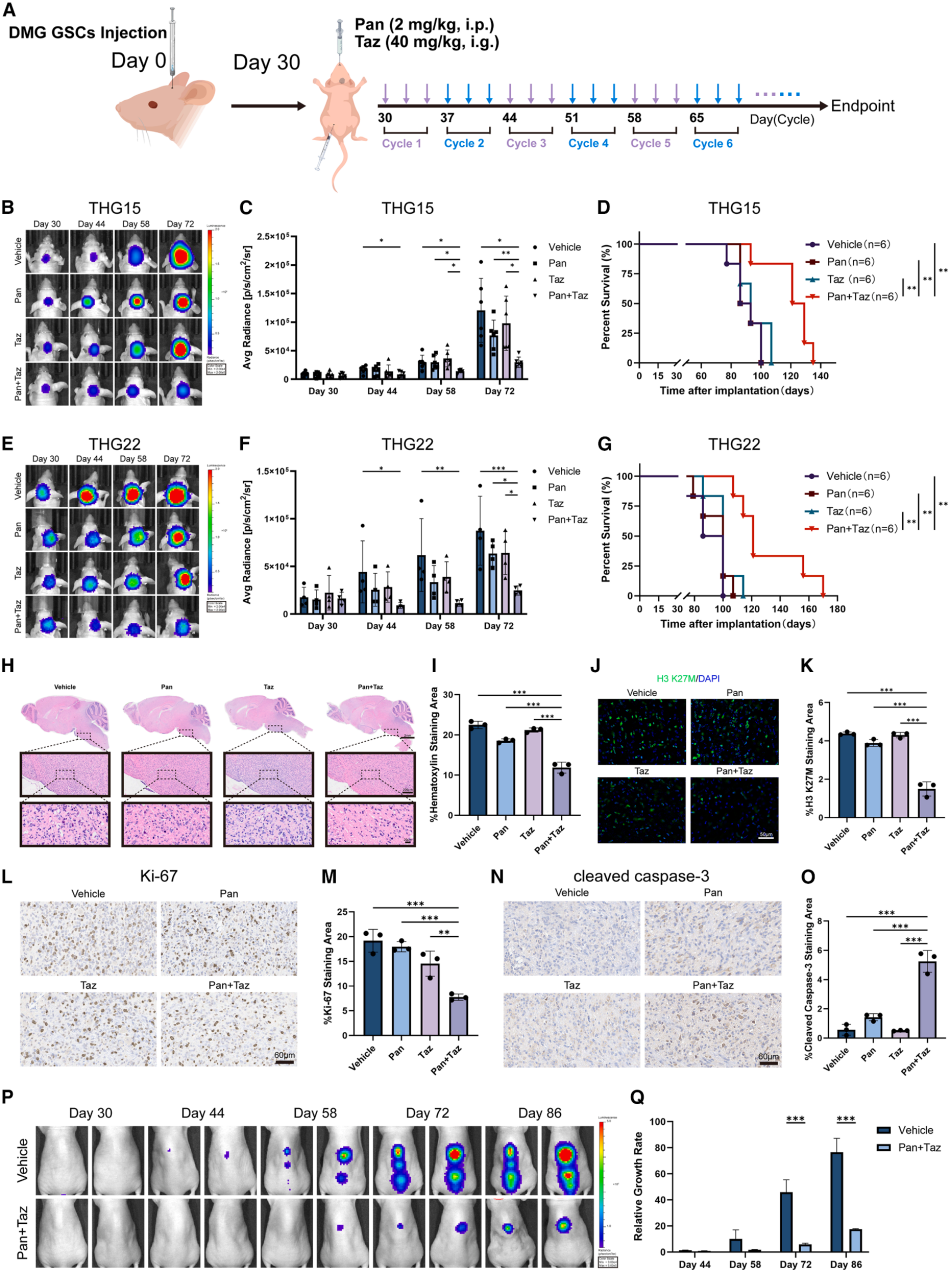
Combined panobinostat and tazemetostat inhibit DMG growth *in vivo* and prolong survival (A) Schematic illustration of the murine xenograft model and drug treatment schedule. Luciferase-labeled DMG GSCs were stereotactically injected into the mouse brainstem on day 0. Tumor establishment was confirmed on day 30, followed by treatment with intraperitoneal (i.p.) panobinostat, intragastric (i.g.) tazemetostat, or their combination, three times a week. (B) Representative *in vivo* bioluminescence images of THG15 GSC-bearing mice at the indicated time points following luciferin administration. (C) Quantification of total tumor burden (average radiance) in THG15 GSC-bearing mice over time. (D) Kaplan-Meier survival curves of THG15 GSC-bearing mice across the indicated treatment groups. Treatment start, day 30. (E) Representative *in vivo* bioluminescence images of THG22 GSC-bearing mice. (F) Quantification of average radiance in THG22 GSC-bearing mice. (G) Kaplan-Meier survival curves of THG22 GSC-bearing mice treated with epigenetic agents. Treatment start, day 30. (H) Representative H&E-stained sections of the brainstem tissues from THG15 GSC-bearing mice following treatment. Scale bars: 2 mm (top), 200 μm (middle), and 20 μm (bottom). (I) Quantification of hematoxylin-positive tumor area shown in (H). For histological quantification, three independent fields of view were analyzed per section from each animal, with three animals included per group. (J) Immunofluorescence staining for the H3K27M mutation protein in the tumors from THG22 GSC-bearing mice. Scale bar, 50 μm. (K) Quantification of H3K27M-positive area shown in (J). (L) Immunohistochemical staining for Ki-67 in tumors of THG15 GSC-bearing mice. Scale bars, 60 μm. (M) Quantification of Ki-67 positive tumor area shown in (L). (N) Immunohistochemical staining for cleaved caspase-3 in tumors from THG15 GSC-bearing mice. Scale bars, 60 μm. (O) Quantification of the cleaved caspase-3 positive area shown in (N). (P and Q) *In vivo* bioluminescence imaging and quantification of spinal dissemination in THG15 GSC-bearing mice. Relative growth rate was calculated as post-treatment signal intensity normalized to pre-treatment signal intensity. Pan, panobinostat; Taz, tazemetostat; ∗*p* < 0.05; ∗∗*p* < 0.01; ∗∗∗*p* < 0.001.

To evaluate *in vivo* efficacy, luciferase-labeled THG15 and THG22 GSCs were orthotopically implanted into the brainstem of BALB/c-nu/nu mice (Figure 3A). Treatment began 30 days after implantation following confirmation of tumor establishment. Therapeutic agents were administered three times weekly (mondays, wednesdays, and fridays) to maintain steady-state drug concentrations until the mice reached humane endpoints. After 2 weeks of therapy (day 44), the combination group showed a markedly reduced tumor burden compared with vehicle or single agents (Figures 3B, 3C, 3E, and 3F). This effect persisted throughout the study: by day 72, tumor volumes in the combination group were approximately one-fourth of those in the vehicle group (Figures 3C and 3F). Combination treatment also significantly extended survival. In THG15-bearing mice, median survival increased to 125 days compared with 89 days (vehicle), 89 days (panobinostat), and 93 days (tazemetostat) (Figure 3D). Similarly, in THG22-bearing mice, median survival reached 121 days versus 93, 100, and 100 days, respectively (Figure 3G). Histopathological analyses after 8 weeks of treatment showed reduced tumor cell density in combination-treated animals (Figures 3H and 3I). H3K27M immunohistochemistry (IHC) further confirmed a marked reduction in mutant tumor cell density under the combination regimen relative to the vehicle group or monotherapies (Figures 3J and 3K). Concurrently, Ki-67 staining revealed a 59.5% decrease in proliferative cells compared with the vehicle group, outperforming either monotherapy (Figures 3L and 3M). Consistently, cleaved caspase-3 staining demonstrated 9.26-, 3.68-, and 10.29-fold elevations in apoptosis relative to the vehicle, panobinostat, and tazemetostat groups, respectively (Figures 3N and 3O). Intriguingly, while untreated THG15 xenografts frequently developed spontaneous spinal dissemination within 4 weeks (Figure 3P), this dissemination was robustly suppressed by the dual-agent therapy (Figures 3P and 3Q). Collectively, these findings demonstrate that combined panobinostat and tazemetostat effectively suppress tumor growth, prolong survival, and reduce spinal dissemination *in vivo*.

### Combined panobinostat and tazemetostat suppress proliferation and induce apoptosis in DMG GSCs

**Figure 4:**
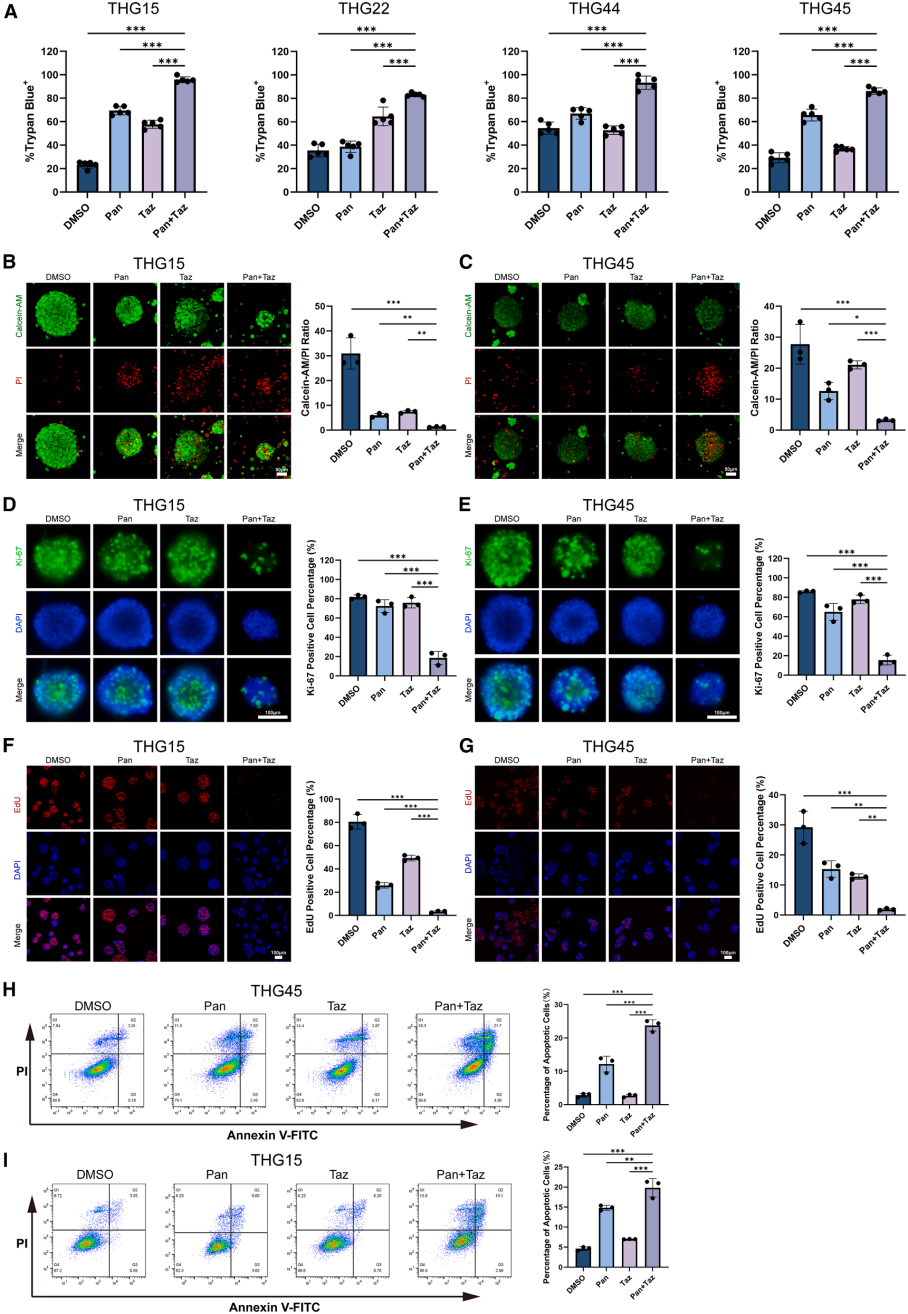
Panobinostat and tazemetostat synergistically inhibit DMG proliferation and induce apoptosis (A) Quantification of trypan blue-positive (nonviable) cells following the indicated drug treatment. (B and C) Representative immunofluorescence images of Calcein AM (green, viable cells) and propidium iodide (PI, red, nonviable cells) staining in epigenetic-agent-treated THG15 (B) and THG45 (C) GSC neurospheres. The ratios of calcein AM-positive area to PI-positive area are quantified on the right. Scale bars, 50 μm. (D and E) Representative immunofluorescence images of the proliferation marker Ki-67 in THG15 (D) and THG45 (E) neurospheres following drug treatment. Nuclei were counterstained with DAPI. Quantification of Ki-67 positive cells is shown on the right. Scale bars, 100 μm. (F and G) Representative EdU incorporation assays indicating proliferating cells in THG15 (F) and THG45 (G) following the indicated drug treatments. DAPI was used to visualize nuclei. Quantification of EdU-positive cells is shown on the right. Scale bars, 100 μm. (H) Flow cytometric analysis of Annexin V-FITC- and PI-stained THG45 GSCs after 7 days of drug treatment. The proportions of Annexin V-positive cells (Q2+Q3) are compared on the right. (I) Flow cytometric analysis of Annexin V-FITC- and PI-stained THG15 GSCs after 5 days of drug treatment. Proportions of Annexin V-positive cells (Q2+Q3) are quantified on the right. Pan, panobinostat; Taz, tazemetostat; ∗*p* < 0.05; ∗∗*p* < 0.01; ∗∗∗*p* < 0.001.

To quantify drug-induced cell death, we first performed trypan blue exclusion assays and observed significantly higher proportions of nonviable cells in the dual-treated group compared with control or single agents across all four GSC cultures (Figure 4A). To avoid dissociation-related artifacts, *in situ* Calcein-AM/propidium iodide (PI) staining was conducted and similarly showed a markedly reduced live-to-dead ratio following combination treatment (Figures 4B, 4C, S3A, and S3B), confirming enhanced cytotoxicity.

We next examined whether decreased viability reflected impaired proliferation or increased apoptosis. Consistent with the sphere-formation data (Figure 2D), Ki-67 immunofluorescence revealed a pronounced reduction in proliferating cells under dual treatment (Figures 4D and 4E). *In vitro* 5-ethynyl-2-deoxyuridine (EdU) incorporation assays further demonstrated near-complete loss of DNA synthesis in combination-treated GSCs, in contrast to robust EdU positivity in controls and single-agent groups (Figures 4F, 4G, S4A, and S4B).

Apoptosis was assessed by flow cytometry after 7 days of treatment. The combination markedly increased apoptotic fractions in THG45, and in THG15 and THG22 substantial apoptotic debris indicated late-stage cell death (Figures 4H, S5A, and S5B). Shortening the treatment to 5 days confirmed consistently elevated apoptosis in the dual-treated groups (Figure 4I). Collectively, these findings demonstrate that combined panobinostat and tazemetostat synergistically suppress proliferation and induce apoptosis in DMG GSCs *in vitro*, consistent with the reduced proliferation and increased apoptosis observed *in vivo*.

### Panobinostat and tazemetostat synergistically induce cell-cycle arrest in DMG GSCs

**Figure 5:**
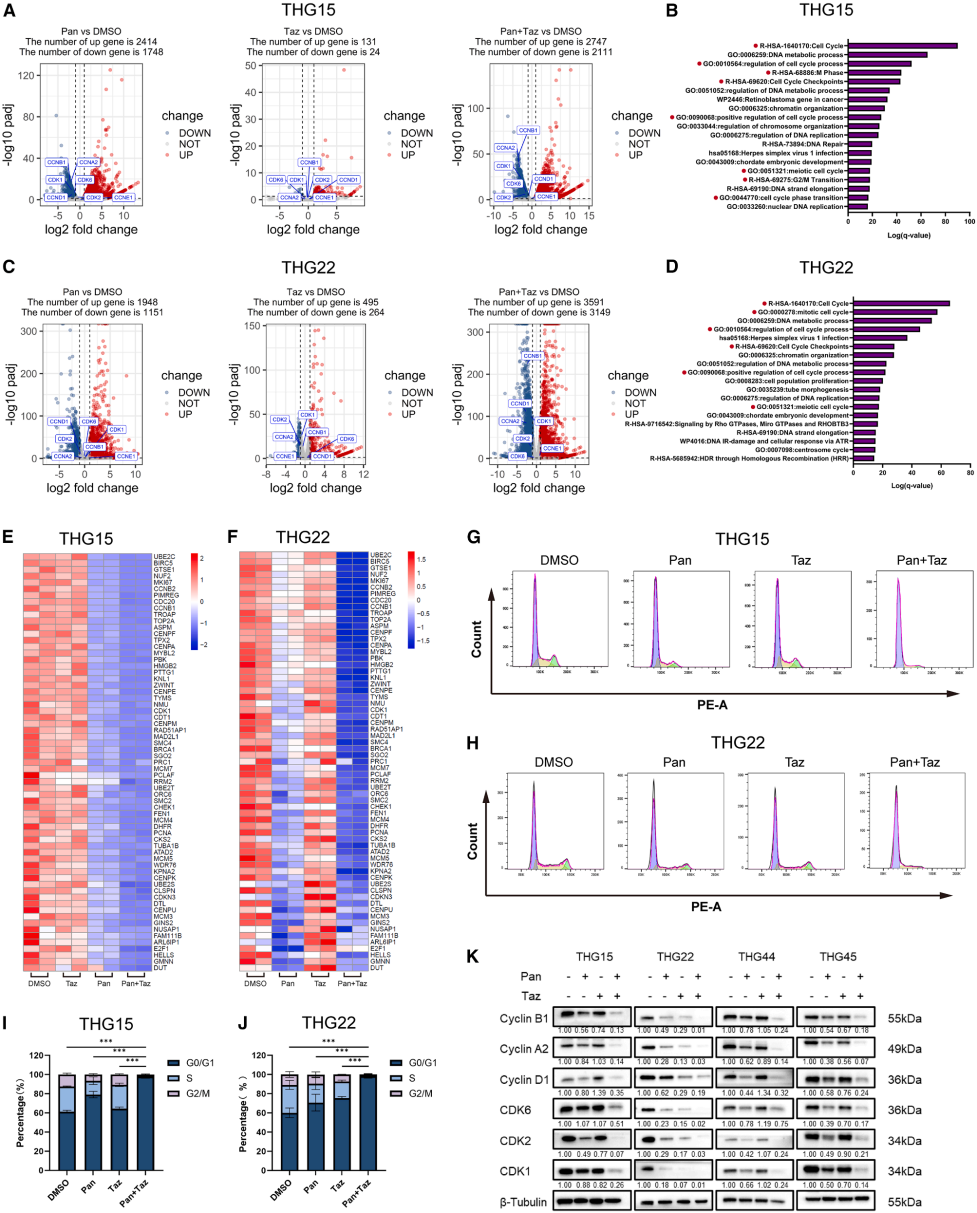
Panobinostat and tazemetostat induce cell-cycle arrest in DMG GSCs (A, C) RNA-seq transcriptomic profiling of THG15 and THG22 GSCs following treatment with DMSO, panobinostat, tazemetostat, or the combination. Differentially expressed genes were defined by |Log_2_ FC| > 1 and an adjusted *p* < 0.05. (B and D) Pathway enrichment analysis of the downregulated genes following the combined treatment in THG15 (B) and THG22 (D) GSCs. (E and F) Heatmaps showing the expression changes of representative cell-cycle-associated genes in THG15 (E) and THG22 (F) GSCs. (G-J) Flow cytometric analysis of cell-cycle distribution in THG15 (G) and THG22 (H) GSCs after the indicated treatments, with statistical significance indicated for the S phase fraction in THG15 (I) and THG22 (J) GSCs. (K) Western blot analysis of cell-cycle-regulated Cyclins and CDKs following 7 days of drug treatment in GSCs. β-tubulin was used as the loading control. Pan, panobinostat; Taz, tazemetostat; ∗*p* < 0.05; ∗∗*p* < 0.01; ∗∗∗*p* < 0.001.

To investigate the transcriptional consequences of dual HDAC/EZH2 inhibition, we performed RNA sequence (RNA-seq) on THG15 and THG22 GSCs after 7 days of treatment. Combination treatment induced extensive transcriptomic alterations (Figures 5A and 5C), with principal component analysis (PCA) showing distinct clustering relative to monotherapies (Figures S6A and S6B). Downregulated genes were significantly enriched in pathways governing cell-cycle progression (Figures 5B and 5D). Consistently, cell cycle-associated genes were uniformly repressed by the combination, whereas single agents produced culture-specific and less consistent effects (Figures 5E and 5F). Flow-cytometric profiling revealed near-complete G0/G1 accumulation after 7 days of dual-agent treatment, with marked depletion of S- or G2/M-phase populations (Figures 5G–5J). Shortening treatment to 5 days produced a partial but significant S-phase reduction (Figure S7A), suggesting a time-dependent inhibition of DNA synthesis. Western blot analyses confirmed marked downregulation of Cyclin A2, Cyclin B1, Cyclin D1, and CDK1/2/6 after 7 days of combination therapy, with weaker effects at 5 days (Figures 5K and S7B).

To further examine histone-mark changes associated with dual treatment, we assessed global H3K27ac and H3K27me3 levels by immunoblotting. Panobinostat-containing treatment increased H3K27ac, consistent with HDAC inhibition, while H3K27me3 showed nonlinear treatment-associated changes rather than a uniform reduction expected from EZH2 inhibition alone (Figure S8). These findings highlight the complexity of H3K27ac/H3K27me3 regulation in H3K27M DMG and indicate that the antitumor effect of dual HDAC/EZH2 inhibition cannot be explained by linear correction of global histone-mark imbalance alone. Accordingly, we interpret the cell-cycle phenotype primarily on the basis of convergent transcriptomic and functional evidence, including repression of cell-cycle genes, G0/G1 accumulation, reduced EdU incorporation, and downregulation of Cyclins and CDKs. Collectively, these findings demonstrate that dual panobinostat/tazemetostat treatment induces coordinated cell-cycle repression and robust G0/G1 arrest in DMG GSCs.

### Combined panobinostat and tazemetostat suppress stemness-associated programs and induce differentiation-associated transcriptional shifts in DMG GSCs

**Figure 6:**
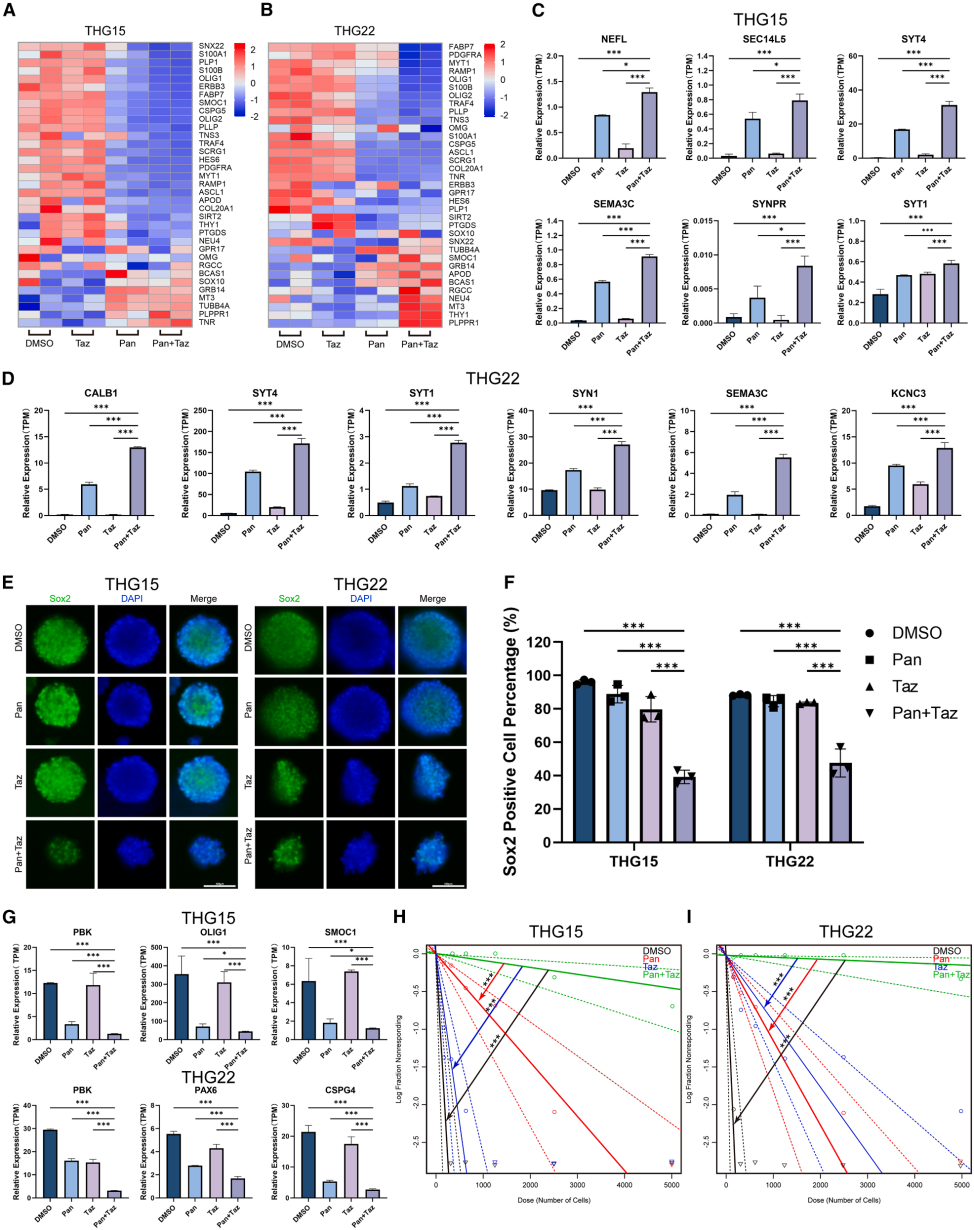
Panobinostat and tazemetostat synergistically suppress stemness-associated programs and induce differentiation-associated transcriptional shifts in DMG GSCs (A and B) RNA-seq analysis showing expression changes of oligodendrocyte progenitor cell (OPC) markers in THG15 (A) and THG22 (B) GSCs after 7 days of the indicated drug treatments. (C and D) Expression levels (measured in transcripts per million, TPM) of differentiation-associated neuronal genes in THG15 (C) (including *NEFL*, *SEC14L5*, *SYT4*, *SEMA3C*, *SYNPR*, and *SYT1*) and THG22 (D) (including *CALB1*, *SYT1*, *SYT4*, *SYN1*, *SEMA3C*, and *KCNC3*) GSCs following the indicated drug treatments. (E) Representative immunofluorescence images of Sox2 expression in THG15 and THG22 GSC neurospheres following the indicated drug treatments. Nuclei were counterstained with DAPI. Scale bars, 100 μm. (F) Quantification of Sox2-positive GSCs from the experiments in (E). (G) RNA-seq analysis showing expression changes of progenitor- and stemness-associated genes, including *PBK*, *OLIG1*, and *SMOC1* in THG15 GSCs, and *PBK*, *PAX6*, and *CSPG4* in THG22 GSCs, following the indicated drug treatments. (H and I) *In vitro* extreme limiting dilution assays (ELDA) demonstrating a reduced self-renewal capacity, as indicated by the increased number of GSCs required to form neurospheres following dual-agent treatment in THG15 (H) and THG22 (I) GSCs. Pan, panobinostat; Taz, tazemetostat; ∗*p* < 0.05; ∗∗*p* < 0.01; ∗∗∗*p* < 0.001.

The H3K27M mutation disrupts PRC2 activity and impedes normal differentiation, maintaining progenitor-like and stem-like programs in DMG GSCs.^24^ Consistent with this biology, transcriptomic profiling revealed that dual-agent treatment led to marked downregulation of OPC-associated markers, including *PDGFRA*, *OLIG1*, and *CSPG5* in both THG15 and THG22 following 7 days of treatment (Figures 6A and 6B). While this repressive effect was largely driven by HDAC inhibition via panobinostat, the dual-agent combination further consolidated these transcriptomic changes. These findings indicate that combined HDAC/EZH2 inhibition more effectively suppresses OPC-like and progenitor-associated programs. Considering that DMG GSCs are transcriptionally heterogeneous and may harbor both OPC-like and neural progenitor cell (NPC)-like programs, and that differentiation blockade across these developmental programs is a hallmark of DMG, we next examined whether dual treatment was associated with differentiation-related transcriptional remodeling. Notably, while panobinostat monotherapy upregulated multiple differentiation-associated neuronal genes, including *NEFL*, *SEC14L5*, *SYT4*, *SEMA3C*, *SYNPR*, and *SYT1*, the combination treatment achieved a substantially more pronounced upregulation of these genes in THG15 (Figure 6C).^25^^,^^26^ A similar neuronal gene-induction pattern was observed in THG22, where *CALB1*, *SYT1*, *SYT4*, *SYN1*, *SEMA3C*, and *KCNC3* were consistently induced (Figure 6D), indicating a reproducible neuronal-like differentiation-associated transcriptional shift. These changes should be interpreted as transcriptional remodeling associated with differentiation programs rather than direct evidence of lineage transdifferentiation from OPC-like cells into neurons.

We then assessed regulators of stemness maintenance. Immunofluorescence staining demonstrated a 46.1%–59.2% reduction in Sox2 expression following combination treatment, significantly greater than reductions observed with either monotherapy (Figures 6E and 6F).^27^ Additional progenitor-associated markers were also significantly downregulated under the dual-treatment regimen. Specifically, expression of *PBK*, *OLIG1*, and *SMOC1* was reduced by 70%–90% in THG15, while a similar substantial reduction in *PBK*, *PAX6*, and *CSPG4* was observed in THG22 (Figure 6G). Functionally, limiting-dilution assays showed a pronounced decrease in sphere-forming capacity with dual treatment (Figures 6H and 6I). Extreme limiting dilution analysis (ELDA) quantification revealed a decline in stem cell frequency from 1/94 to 1/10,900 (95% confidence interval [CI]: 1/23,982–1/4,953) in THG15 and from 1/70 to 1/36,820 (95% CI: 1/145,105–1/9,343) in THG22, corresponding to reductions of 91.4% and >99%, respectively. Collectively, these findings demonstrate that dual panobinostat/tazemetostat treatment suppresses OPC-like and progenitor-associated programs, induces differentiation-associated neuronal gene expression, and substantially reduces the self-renewing stem-like compartment in DMG GSCs.

## Discussion

H3K27-altered DMG is primarily driven by epigenetic dysregulation arising from oncohistone-mediated disruption of PRC2 function, rendering chromatin-modulating agents a biologically compelling therapeutic approach. Panobinostat, a pan-HDAC inhibitor approved for relapsed multiple myeloma, demonstrates a favorable multiparameter optimization score, indicating improved blood-brain barrier penetrance compared to other epigenetic modulators.^28^ Tazemetostat, a selective EZH2 inhibitor approved for epithelioid sarcoma and follicular lymphoma, directly targets the catalytic subunit of PRC2. Preclinical studies have shown that it reduces viability and proliferation of H3K27M-mutant glioma cells by derepressing silenced tumor suppressors such as *CDKN2A*.^9^ However, epigenetic monotherapies have thus far yielded limited clinical efficacy in glioma. In adults with recurrent high-grade glioma, panobinostat in combination with bevacizumab failed to improve 6-month progression-free survival,^29^ and similarly, the combination of vorinostat (another HDAC inhibitor) and bevacizumab was not superior to bevacizumab alone in terms of progression-free survival or overall survival in a randomized phase 2 trial for recurrent glioblastoma.^30^ In pediatric diffuse intrinsic pontine glioma (DIPG), the Pediatric Brain Tumor Consortium (PBTC-047) phase 1 study established the safety profile of panobinostat but observed no clear evidence of survival benefit, with median progression-free survival and overall survival comparable to historical controls.^31^ To date, tazemetostat has not been clinically evaluated in glioma beyond our own exploratory trial. These collective findings highlight the inadequacy of epigenetic monotherapy in DMG and reinforce the need to explore mechanistically rational combinations with synergistic potential.

In this context, our previous clinical study explored the combination of panobinostat and tazemetostat as the epigenetic backbone of an immunotherapeutic regimen. Encouraging responses, including extended survival in patients with relapsed and leptomeningeal DMG, suggest that dual inhibition of HDAC/EZH2 may overcome limitations of monotherapy. Although our previous work suggested that these agents may induce epigenetic reprogramming and suppress proliferation- and stemness-associated pathways in DMG GSC,^15^^,^^23^ the synergistic effect of panobinostat and tazemetostat and the molecular mechanisms underlying this interaction in DMG have not been elucidated, which is the central focus of the present study.

In this study, we provide the first preclinical evidence that dual HDAC/EZH2 inhibition produces a robust and reproducible synergistic antitumor effect not achievable by either agent alone in DMG, across four patient-derived GSC cultures originating from both brainstem and spinal cord DMG lesions. The dual-agent regimen consistently suppressed GSC growth, proliferation, and self-renewal capacity, promoted apoptosis, induced cell-cycle arrest and differentiation-associated transcriptional remodeling, and significantly reduced tumor burden and spinal dissemination while prolonging survival in orthotopic xenografts. This cross-model consistency addresses a long-standing challenge in DMG preclinical research, where therapeutic effects often fail to generalize across anatomically or genetically distinct patient-derived models.^24^

Mechanistically, our data identify cell-cycle arrest as a central vulnerability engaged by the dual regimen. Transcriptomic profiling consistently showed downregulation of key cell-cycle regulators, including multiple Cyclins and CDKs, accompanied by near-complete G0/G1 arrest and loss of S-phase cells. While HDAC or EZH2 inhibition alone has been reported to only partially suppress proliferation in H3K27M gliomas,^9^^,^^28^^,^^32^^,^^33^ the combination elicited a coordinated shutdown of cell-cycle progression across patient-derived DMG GSC models.

An important implication of these findings is that the response to dual HDAC/EZH2 inhibition does not simply reflect restoration of the canonical H3K27M-associated imbalance between elevated H3K27ac and reduced H3K27me3 levels.^9^^,^^32^ Panobinostat increased H3K27ac, as expected from HDAC inhibition. However, despite this increase in a histone mark generally associated with transcriptional activation, the dual treatment still produced marked repression of cell-cycle programs and robust G0/G1 arrest. This apparent paradox suggests that the therapeutic effect is better understood as a network-level disruption of epigenetically maintained proliferative and stem-like programs, rather than as a simple correction of a single histone modification abnormality. The precise locus-specific chromatin mechanisms underlying this response warrant further investigation using dedicated epigenomic approaches, such as chromatin immunoprecipitation sequencing (ChIP-seq), cleavage under target and tagmentation (CUT&Tag), and assay for transposase-accessible chromatin using sequencing (ATAC-seq).

In addition to enforcing cell-cycle arrest, the dual regimen reshaped the developmental identity of DMG GSCs in a manner consistent with current models of H3K27M gliomagenesis. OPC-like transcriptional programs are widely considered a putative cell-of-origin and a key driver of proliferation and invasion in H3K27M tumors,^24^ a concept supported by murine and induced pluripotent stem cell (iPSC)-derived models in which H3K27M cooperates with *PDGFRA* activation or *Trp53*/*PTEN* loss in OPC-lineage cells.^34^^,^^35^^,^^36^^,^^37^^,^^38^ Within this framework, the consistent downregulation of OPC-associated markers, including *PDGFRA*, *OLIG1*, and *CSPG5*, after dual HDAC/EZH2 inhibition indicates disruption of lineage programs that sustain the OPC-like state in DMG. This loss of OPC signatures, together with the induction of differentiation-associated genes, suggests differentiation-associated transcriptional remodeling rather than definitive lineage conversion. The convergence of OPC program suppression and differentiation-gene induction implies that dual inhibition perturbs the progenitor-like and stem-like programs on which DMG GSCs depend for proliferation and invasion. Comparable differentiation-associated responses have been reported in other epigenetic interventions, such as dual HDAC/LSD1 inhibition driving chromatin reprogramming and a differentiation phenotype in DIPG.^25^ Our data extend this paradigm to H3K27M DMG, supporting a model in which dual HDAC/EZH2 inhibition suppresses OPC-like progenitor programs and induces differentiation-associated transcriptional remodeling, rather than proving direct lineage conversion.

Functionally, these lineage and transcriptional shifts were accompanied by a marked reduction in the self-renewing GSC compartment. While reductions in stemness markers are commonly reported in epigenetic studies, our limiting-dilution assays provide quantitative evidence that the self-renewing fraction declined by 91.4% and >99% in THG15 and THG22, respectively. Given the central role of GSCs in treatment resistance, recurrence, and dissemination,^39^ this marked reduction in self-renewing capacity provides a plausible biological explanation for both the durable tumor control and reduced spinal dissemination observed *in vivo*. The precise mechanisms linking epigenetic co-targeting to impaired spinal dissemination remain to be fully elucidated and warrant further investigation.

Taken together, our work defines a unified mechanistic framework in which dual HDAC/EZH2 inhibition functions as a multidimensional epigenetic strategy for DMG. These integrated effects not only account for the encouraging clinical responses previously observed with this therapeutic backbone but also provide a strong rationale for incorporating this approach into future therapeutic regimens, particularly as a backbone for combination with immunotherapy. More broadly, our findings suggest that in a tumor characterized by abnormally elevated H3K27ac and markedly reduced H3K27me3, simultaneous perturbation of HDAC- and EZH2- regulated programs does not restore the canonical epigenetic balance, but instead further rewires the dysregulated epigenetic landscape to destabilize proliferative and stem-like programs that sustain tumor growth. This conceptual framework may shed light on the development of rational epigenetic strategies for DMG and other malignancies driven by aberrant epigenetic dysregulation.

## Materials and methods

### Patient-derived cell cultures

Surgical tumor specimens were obtained from DMG patients with informed consent in accordance with the ethical guidelines approved by the Ethics Committee of Beijing Tsinghua Changgung Hospital. Patient-derived DMG GSC cultures were established as previously described,^40^ and were designated as THG 15, 17, 22, 44, and 45, with no evidence of human cell-line cross-contamination (Table S4). Briefly, mechanically minced tumor tissues were treated with 0.25% trypsin (Gibco, Waltham, MA, USA) and 10 U/mL DNase I (Roche, Mannheim, Germany), and were subsequently triturated and filtered through a 100 μm cell strainer following red blood cell lysis and several washing steps. The filtered GSCs were then plated in EF medium, consisting of Neurobasal medium (Gibco), 3 mM L-glutamine (Sigma-Aldrich, St. Louis, MO, USA), 1× B27 supplement (Gibco), 0.5 × N2 supplement (Gibco), 2 μg/mL heparin (Sigma-Aldrich), 20 ng/mL recombinant human epidermal growth factor (EGF, Sigma-Aldrich), 20 ng/mL recombinant human FGF2 (Sigma-Aldrich), and 0.5× Penicillin-Streptomycin (Gibco). Neurosphere passaging was carried out through dissociation using the NeuroCult Chemical Dissociation Kit (STEMCELL Technologies, Vancouver, BC, Canada).

### Cell viability assay and synergy analysis

Panobinostat (Selleck, Houston, TX, USA) and tazemetostat (Selleck, Houston, TX, USA) were dissolved in DMSO and serially diluted in EF medium. Patient-derived GSCs were plated in 96-well plates at a density of 2 × 10^4^ cells per well. Twenty-four hours after plating, GSCs were treated with either drug alone or a combination of both for 7 days. For the checkerboard co-treatment design, panobinostat (at concentrations of 0, 1, 5, 10, 20, and 50 nM) and tazemetostat (at concentrations of 0, 5, 10, 25, 35, and 50 μM) were combined in all possible pairwise combinations (a 6 × 6 matrix). Cell viability was determined using the CCK-8 assay (Dojindo Molecular Technologies, Kumamoto, Japan) according to the manufacturer’s protocol. The dose-response curves were generated using GraphPad Prism 10. The synergistic effect was analyzed using SynergyFinder 3.0.^41^ A ZIP synergy score greater than 10 was indicative of a synergistic effect. All assays were performed with three independent biological replicates (*n* = 3).

### Sphere formation assay

A total of 2 × 10^5^ DMG GSCs per well were seeded into 6-well plates and treated with either panobinostat or tazemetostat alone or both 24 h after plating. On day 7 post-treatment, the diameter and number of neurospheres were determined via microscopic imaging using a Nikon microscope and analyzed with ImageJ. Neurospheres with a diameter exceeding 100 μm were considered positive tumor spheres with stable sphere-forming ability.

### Patient-derived xenograft in a murine model

The animal study was approved by the Institutional Animal Care and Use Committee of the Laboratory Animal Resources Center at Tsinghua University and was conducted in strict accordance with the guidelines. The patient-derived xenograft model utilized 4-week-old female BALB/C-nu/nu mice, purchased from Charles River Laboratories (Beijing, China) and housed in a pathogen-free environment. Approximately, 5 × 10^5^ DMG GSCs infected with lentivirus carrying the luciferase sequence were injected into the brainstem of the mice, with the injection site located 1.0 mm posterior to the Lambda point, 0.8 mm to the right, and at a depth of 4.6 mm. Thirty days after the injection, the tumor growth was assessed through *in vivo* bioluminescence imaging following intraperitoneal (i.p.) injection of D-luciferin potassium (Yeasen, Shanghai, China). Based on the signal intensity, the tumor-bearing mice were stratified and randomly assigned to either the vehicle group or the treatment group. In the treatment group, panobinostat (2 mg/kg) was administered via i.p. injections, and tazemetostat (40 mg/kg) was administered by intragastric injections (i.g.), three times a week (e.g., monday, wednesday, and friday). The vehicle group received the same volume of solvent. The pharmacological treatment was initiated on day 30 post-implantation and administered continuously until the humane endpoint of each animal was reached. The mice were sacrificed to obtain tumor tissues for pathologic analysis.

### Cell viability quantification

DMG GSC viability was quantified using the trypan blue exclusion assay. GSCs were first dissociated into single-cell suspensions and subsequently resuspended in the trypan blue staining buffer (Invitrogen, Waltham, MA, USA). Stained GSCs were manually counted under a light microscope. To minimize the potential interference caused by the cell dissociation process, a Calcein AM/PI Double Staining Kit (Elabscience, Wuhan, China) was also utilized. Briefly, GSCs were incubated with a pre-mixed solution of Calcein AM and PI for 30 min, following the manufacturer’s protocol. Fluorescence signals were subsequently imaged with Nikon confocal microscopy and analyzed on ImageJ.

### Immunofluorescence staining

GSCs were fixed with 4% paraformaldehyde (PFA) for 20 min, followed by permeabilization with 0.5% Triton X-100 (Beyotime, Shanghai, China) for 20 min. Blocking was performed with 5% BSA (Solarbio, Beijing, China) at room temperature for 30 min. Incubation with the primary antibodies was conducted overnight at 4°C, followed by incubation with the secondary antibodies at room temperature for 1 h, shielded from light. Detailed information about the antibodies is provided in Table S3. DAPI staining was conducted at room temperature for 5 min, also shielded from light. Fluorescence detection was performed using a Nikon fluorescence microscope analyzed with ImageJ.

### EdU cell proliferation assay

Cell proliferation was assessed using the EdU Cell Proliferation Kit with Alexa Fluor 647 (Epizyme, Shanghai, China). Specifically, DMSO- or drug-treated DMG GSCs were incubated with the EdU reagent for 4 h, followed by immediate fixation and permeabilization. Fluorophore labeling was performed using the click reaction solution from the kit. Cell nuclei were stained with DAPI (Solarbio). Fluorescence analysis was performed through imaging under a Nikon confocal microscope analyzed with ImageJ.

### Flow cytometry

Cell cycle analysis was conducted utilizing the Cell Cycle Assay Kit (Dojindo Molecular Technologies). Briefly, GSCs were dissociated into a single-cell suspension and subsequently fixed overnight at −20°C with 70% ethanol. After ethanol removal, the cells were incubated with a working solution containing the PI Solution and RNase Solution for 1 h in the dark. Apoptosis assessment was performed using the Annexin V-FITC/PI Apoptosis Detection Kit (Dojindo Molecular Technologies). GSCs were dissociated into a single-cell suspension and resuspended in the Annexin V binding buffer. Annexin V-FITC conjugate and PI Solution were sequentially introduced, followed by incubation at room temperature in the dark for 15 min. Samples were analyzed using the LSRFortessa flow cytometer.

### RNA-seq and analysis

RNA-seq was independently performed for the present study using THG15 and THG22 GSCs harvested 7 days after treatment with the DMSO, panobinostat, tazemetostat, or the combination. RNA sequence reads were aligned to the human reference genome GRCh38 using HISAT2 (v.2.2.1) with the default parameters. The coding gene annotation of hg38 was downloaded from ENSEMBL. For downstream expression analysis, the gene-level count data were extracted from the mapped HISAT2 output using featureCounts (v.2.0.3). Transcripts per kilobase million (TPM), calculated from counts of all samples, were used to determine the expression levels using TPMCalculator. Differential expression analysis using two biological replicates per condition was performed with the DESeq2 R package (1.36.0). DESeq2 provided statistical routines for determining differential expression in digital gene expression data using a model based on the negative binomial distribution. *p* values were adjusted using false discovery rate (FDR) correction. DEGs were defined according to the cutoff criteria: FDR <0.05 and |Log2 FC| > 1.

### Western blot

Cells were lysed on ice for 20 min using the Western/IP lysis buffer (containing a 50× protease/phosphatase inhibitor cocktail; Beyotime, Shanghai, China). After centrifugation, the supernatant was collected and mixed with the loading buffer (Epizyme), followed by boiling at 100°C for 10 min. Protein samples were separated by 10% or 12.5% SDS-PAGE gel electrophoresis and then transferred to a PVDF membrane (Millipore, Billerica, MA, USA). The blots were blocked with 5% skim milk (Solarbio) at room temperature for 1 h. Incubation with the primary antibodies was conducted overnight at 4°C, followed by incubation with the secondary antibody at room temperature for 1 h. Detailed information about the antibodies is provided in Table S3. The blots were treated with the enhanced chemiluminescence (ECL) chemiluminescent reagent (Yeasen) and the signal acquisition was conducted with a chemiluminescence imaging system (Tanon, Shanghai, China).

### Limiting dilution assay

DMSO- or drug-treated GSC neurospheres were dissociated into single cells and resuspended in culture medium. The cells were then seeded into a 96-well plate with a density gradient of 5,000, 2,500, 1,250, 625, 312, and 156 cells per well. After culturing for 10 days, the number of positive wells containing spheroids of >100 μm in diameter was counted. The subsequent analysis was performed using the ELDA online platform (http://bioinf.wehi.edu.au/software/elda/).^42^

### Statistical analysis

Data analysis was performed using GraphPad Prism 10. Intergroup comparisons between two independent samples were conducted using two-tailed unpaired Student’s *t* tests, while multiple group comparisons were analyzed via one-way ANOVA. Survival curve differences were assessed by log-rank tests. A threshold of *p* < 0.05 was considered statistically significant. Statistical significance is indicated as follows: ∗*p* < 0.05; ∗∗*p* < 0.01; ∗∗∗*p* < 0.001; and, n.s., not significant. All data are presented as mean ± standard error of the mean (mean ± SEM).

## Data and code availability

Data will be made available upon request by email.

## Acknowledgments

We thank Prof. Songhai Shi, Hugo Shong, and Dr. James Jin Wang for their academic support, encouragement, and inspiration. We thank Prof. Haitai Li and Deng Pan from the School of Basic Medical Sciences, Tsinghua University, Prof. Da Mi and Kun Li from the School of Life Sciences, Tsinghua University, and Prof. Chunxia Zhang from the Institute of Genetics and Developmental Biology, Chinese Academy of Sciences for their constructive suggestions. We thank Dr. Guihuai Wang and Linkai Jing from the Department of Neurosurgery, Beijing Tsinghua Changgung Hospital, for obtaining the tumor samples. This work was supported by the Science and Technology Innovation Development Project, 10.13039/501100012425Beijing Children's Hospital, Capital Medical University, National Center for Children’s Health (no. YN202504), Tsinghua University-Toyota Joint Research Fund Program (no. 20253930040), the 10.13039/501100001809National Natural Science Foundation of China grants (no. 82503964), 10.13039/501100004772Ningxia Natural Science Foundation of China (no. 2026AAC031081), and the Ningxia Young Elite Scientists Sponsorship Program.

## Author contributions

Conceptualization, Z.Z., Q.G., and W.Z.; project administration, Z.Z. and W.Z.; methodology, Z.Z., Q.G., Y.A., and Y.D.; investigation, Z.Z., Q.G., Y.A., Y.S., and W.Z.; data curation, Z.Z., Q.G., Y.S., and S.C.; funding acquisition, W.Z. and X.N.; visualization, Z.Z. and Y.S.; writing – original draft, Z.Z. and Z.Q.; writing – review and editing, W.Z. and X.N. All authors read and approved the final manuscript.

## Declaration of interests

The authors declare no conflict of interest.

## Supplemental information

Document S1. Figures S1–S8 and Tables S1–S3

Table S4. Genotyping of short tandem repeat and Amelogenin loci in GSCs

Document S2. Article plus supplemental information
