# Supplementary material for: Synergistic cell-cycle repression by HDAC/EZH2 co-targeting in H3K27-altered diffuse midline glioma
Source: Mol Ther Oncol. 2026 Jul 24;34(3):201306. doi: 10.1016/j.omton.2026.201306 (PMC13476686; doi:10.1016/j.omton.2026.201306)
Supplement: Supplementary file 1 [file mmc1.pdf]

OMTON, Volume *34*

Supplemental information

Synergistic cell-cycle repression by HDAC/EZH2 co-targeting in H3K27-altered diffuse midline glioma

Zili Zhen, Qiang Gao, Yong Ai, Zhihong Qian, Yukui Shang, Yafang Deng, Shuai Chen, Xin Ni, and Wei Zhang

Table S1 Clinical information regarding the sources of DMG samples

Cells	Age (Years)	Gender	Tumor Location	H3.3 K27M	Previous Treatment
THG15	28	Male	Spinal cord	Positive	Surgery only
THG17	24	Male	Spinal cord	Positive	None
THG22	14	Female	Spinal cord	Positive	None
THG44	54	Male	Brainstem	Positive	None
THG45	15	Male	Brainstem	Positive	None

Table S2 Drug concentrations to achieve the optimal synergistic effect

Cells	Concentration of Panobinostat	Concentration of Tazemetostat	Average Synergy Score
THG15	10 nM	25 μM	15.737
THG22	10 nM	25 μM	21.871
THG44	5 nM	10 μM	22.016
THG45	10 nM	25 μM	14.9

Table S3 Detailed information of the antibodies

Antibodies	Company	Catalog No.	Dilution	Purpose
CDK1	Proteintech	19532-1-AP	1:1000	Western blot
CDK2	Abcam	ab32147	1:10000	Western blot
CDK6	Abcam	ab124821	1:2000	Western blot
Cyclin B1	Abcam	ab181593	1:2000	Western blot
Cyclin D1	Abcam	ab134175	1:10000	Western blot
Cyclin A2	Abcam	ab211735	1:2000	Western blot
Histone H3	Abcam	ab1791	1:10000	Western blot
H3K27me3	ABclonal	A25096	1:5000	Western blot
H3K27ac	CST	8173S	1:2000	Western blot
Histone H3 (mutated K27M)	Abcam	ab190631	1:200	Immunofluorescence
Sox2	CST	23064	1:200	Immunofluorescence
CD133	ABclonal	A0219	1:200	Immunofluorescence
Nestin	ABclonal	A11861	1:200	Immunofluorescence
PDGFRα	ABclonal	A26747	1:200	Immunofluorescence
Ki-67	ABclonal	A20018	1:200	Immunofluorescence
Cleaved caspase3	CST	9661	1:400	Immunohistochemistry
β-Tubulin	ABclonal	AC021	1:10000	Western blot
HRP Goat mouse IgG (H+L)	ABclonal	AS003	1:10000	Western blot
HRP Goat anti-Rabbit IgG (H+L)	ABclonal	AS014	1:10000	Western blot
FITC Goat anti-Rabbit IgG (H+L)	ABclonal	AS011	1:200	Immunofluorescence
FITC Goat anti-Mouse IgG (H+L)	Invitrogen	A32731	1:200	Immunofluorescence

Table S4 Genotyping of short tandem repeat and Amelogenin loci in GSCs

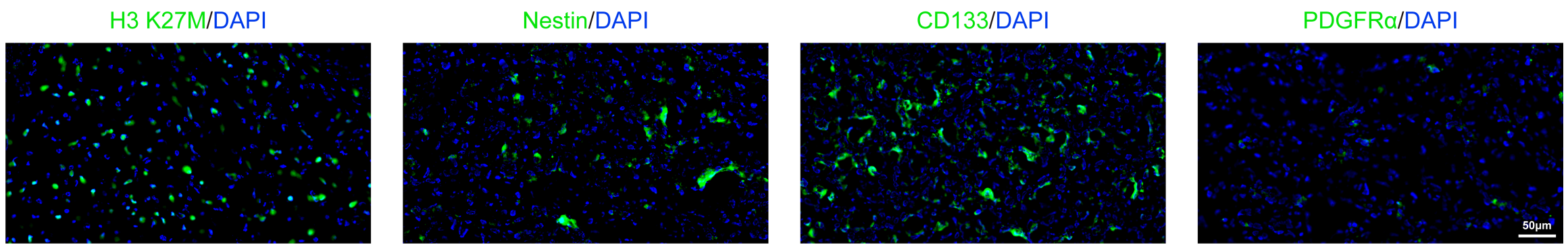

Figure S1 Representative immunofluorescence staining of THG15-derived xenograft tumors. Representative immunofluorescence images of THG15-derived xenograft tumor sections stained for the mutant histone H3K27M, neural stem cell marker Nestin, cancer stem cell marker CD133, and oligodendrocyte progenitor cell marker PDGFRα. Nuclei were counterstained with DAPI (blue). Scale bar: 50 μm.

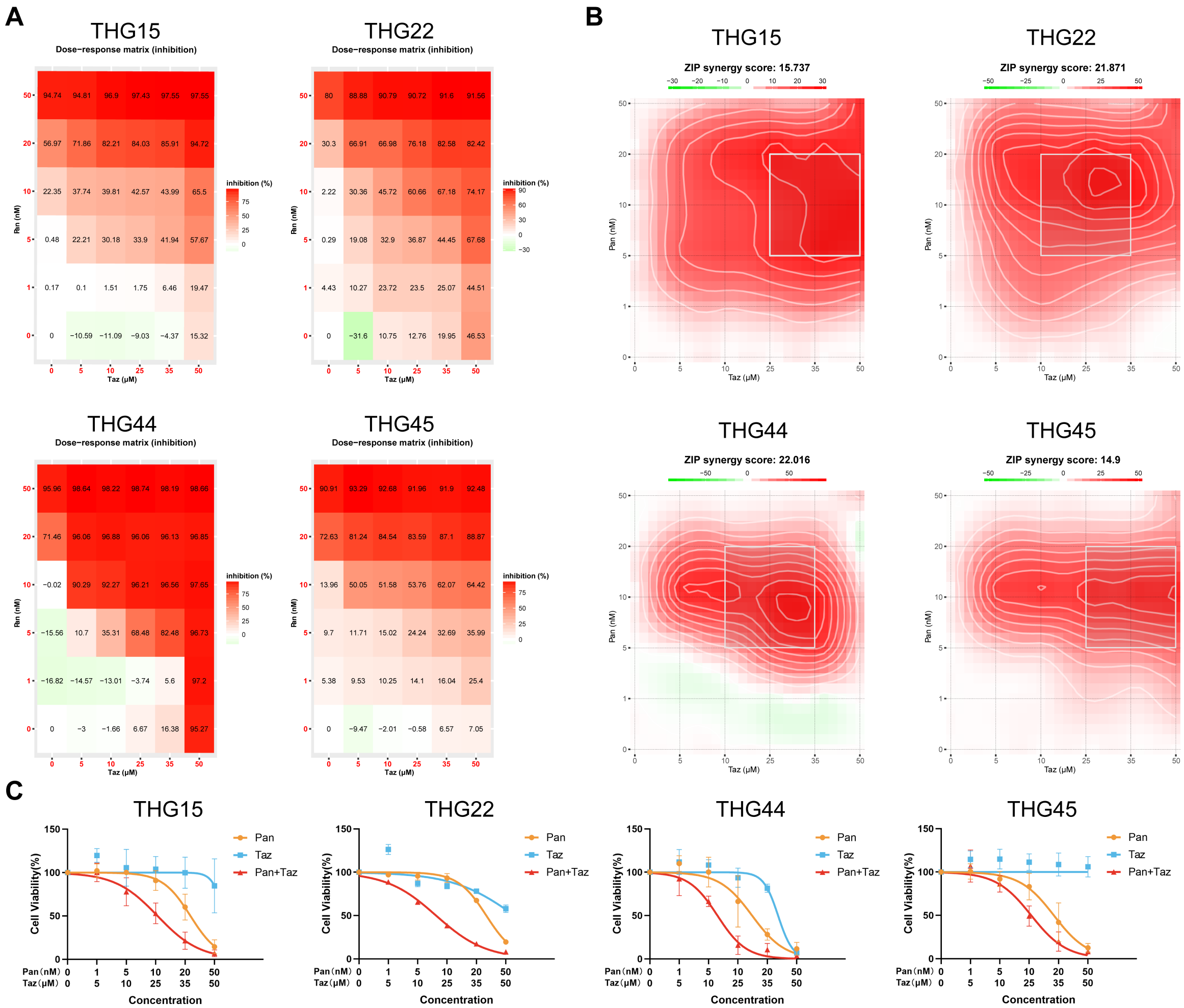

Figure S2 Synergistic inhibitory effects of panobinostat and tazemetostat on DMG GSC growth. (A) Inhibition rate matrices for the combined treatment with panobinostat and tazemetostat in DMG GSC cultures. (B) Three-dimensional synergy landscapes illustrating the synergistic inhibitory effect of panobinostat and tazemetostat. (C) Comparison of cell viability inhibition rates between monotherapies (panobinostat or tazemetostat alone) and the combination therapy, demonstrating that the combined treatment significantly enhances the inhibition of DMG GSC viability compared to either drug administered individually. Pan, panobinostat; Taz, tazemetostat.

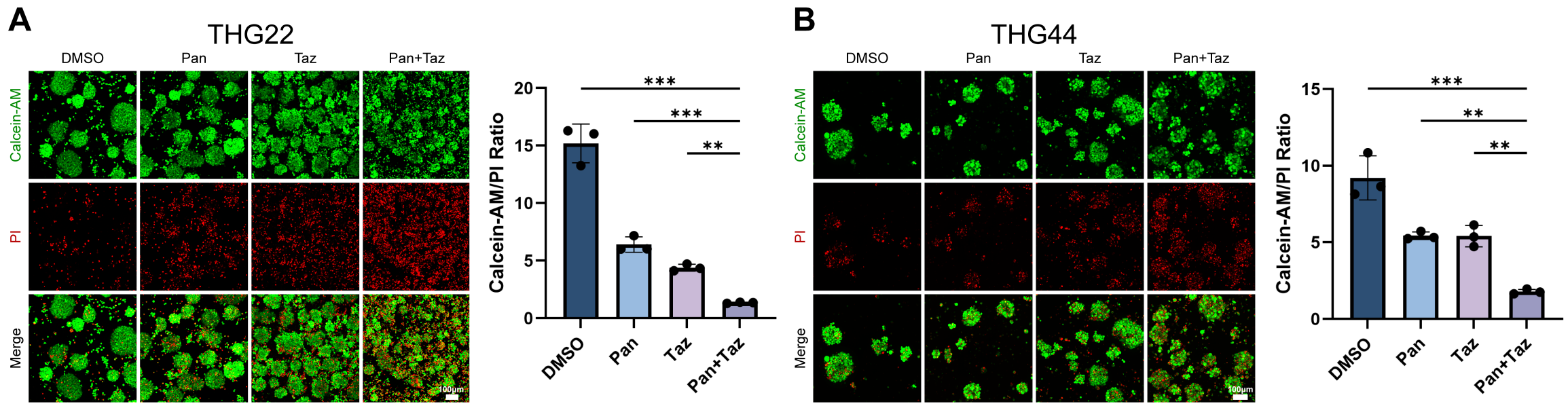

Figure S3 Increased DMG GSC cell death following dual-agent treatment. Representative immunofluorescence images for Calcein AM (green, viable cells) and propidium iodide (PI, red, nonviable cells) in epigenetic agent-treated THG22 (A) and THG44 (B) GSC neurospheres. Ratios of Calcein AM-positive area to PI-positive area are shown on the right. Scale bars, 100 μm. Pan, panobinostat; Taz, tazemetostat; *, P<0.05; **, P<0.01; ***, P<0.001.

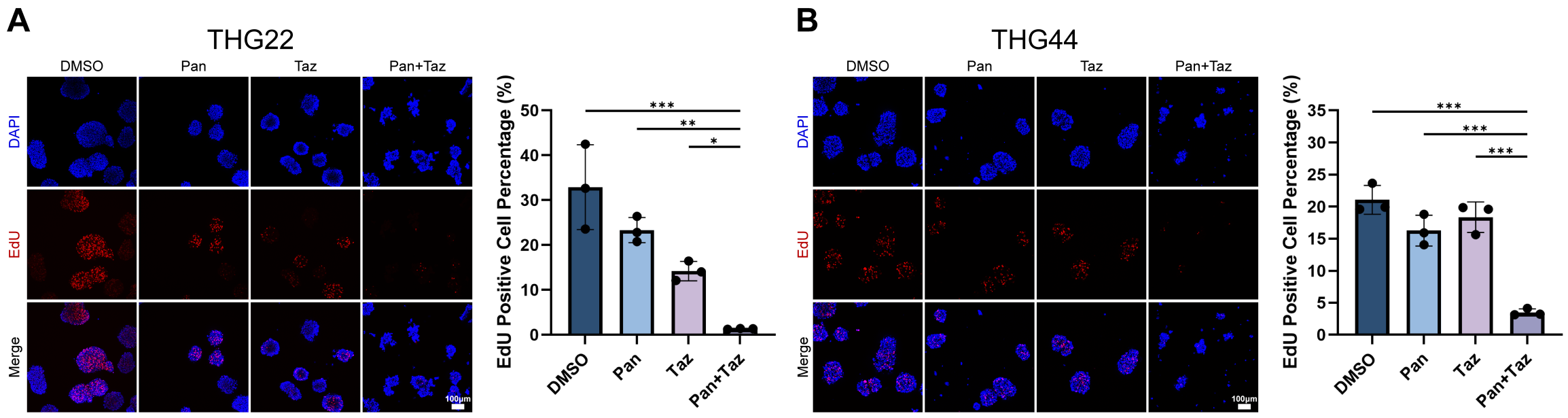

Figure S4 Decreased DMG GSC cell proliferation following dual-agent treatment. Representative immunofluorescence images of EdU incorporation indicating proliferating cells in THG22 (A) and THG44 (B) following drug treatment. DAPI was used to visualize nuclei. Quantification of EdU-positive cells is shown on the right. Scale bar, 100 μm. Pan, panobinostat; Taz, tazemetostat; *, P<0.05; **, P<0.01; ***, P<0.001.

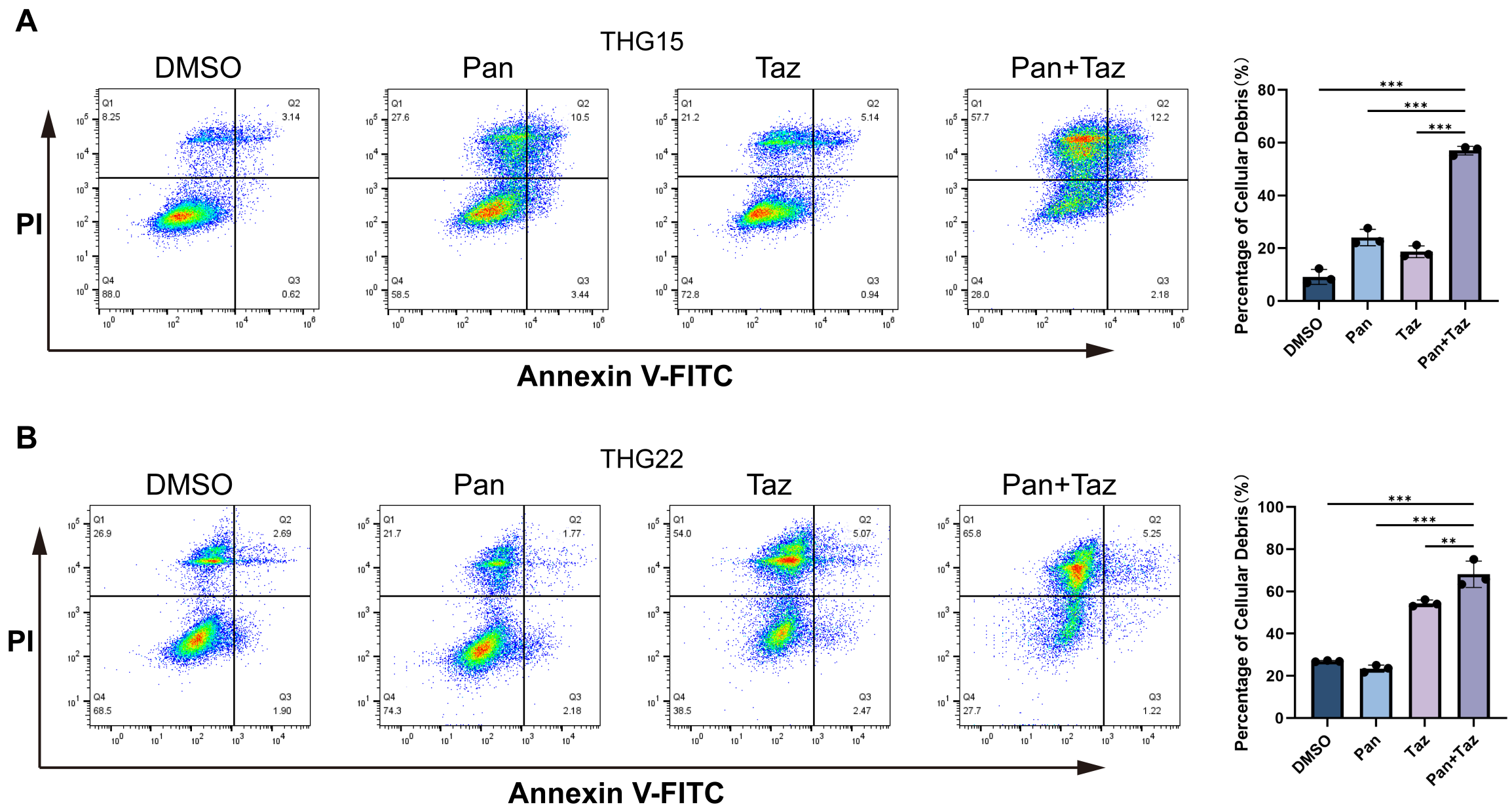

Figure S5 Apoptotic changes and cell death in DMG GSCs following drug treatment. Flow cytometry analysis of Annexin V-FITC and PI staining in THG15 (A) and THG22 (B) GSCs after 7 days of drug treatment. Proportions of cellular debris (Q1 quadrant) are quantified on the right. Pan, panobinostat; Taz, tazemetostat; *, P<0.05; **, P<0.01; ***, P<0.001.

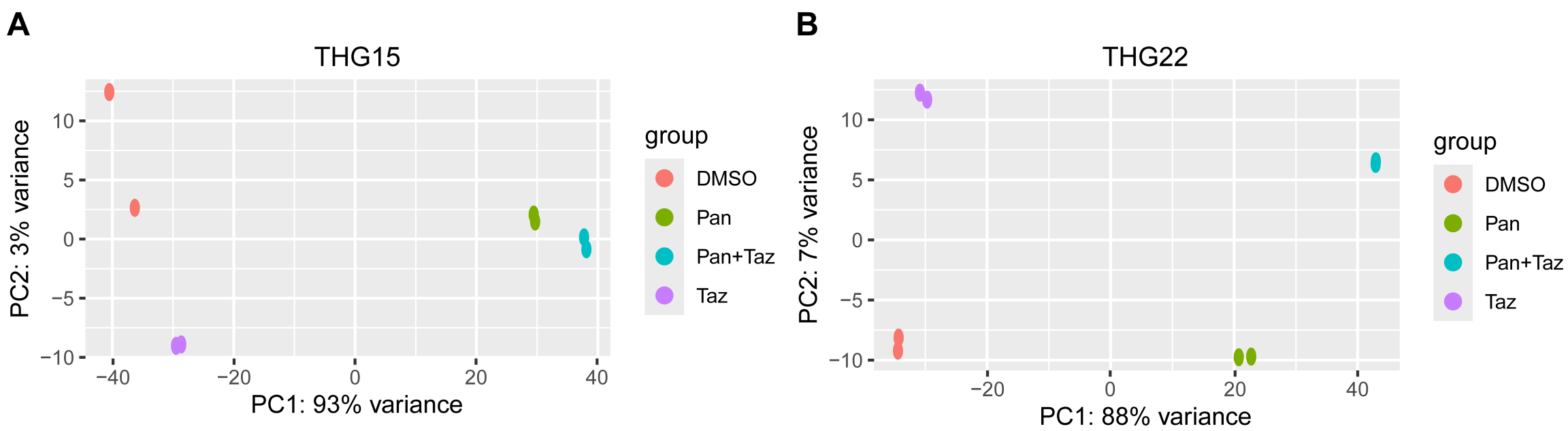

Figure S6 Principal component analysis of transcriptomic profiles in DMG GSCs following drug treatment. Principal component analysis (PCA) of transcriptomic profiles in THG15 (A) and THG22 (B) GSCs following drug treatment.

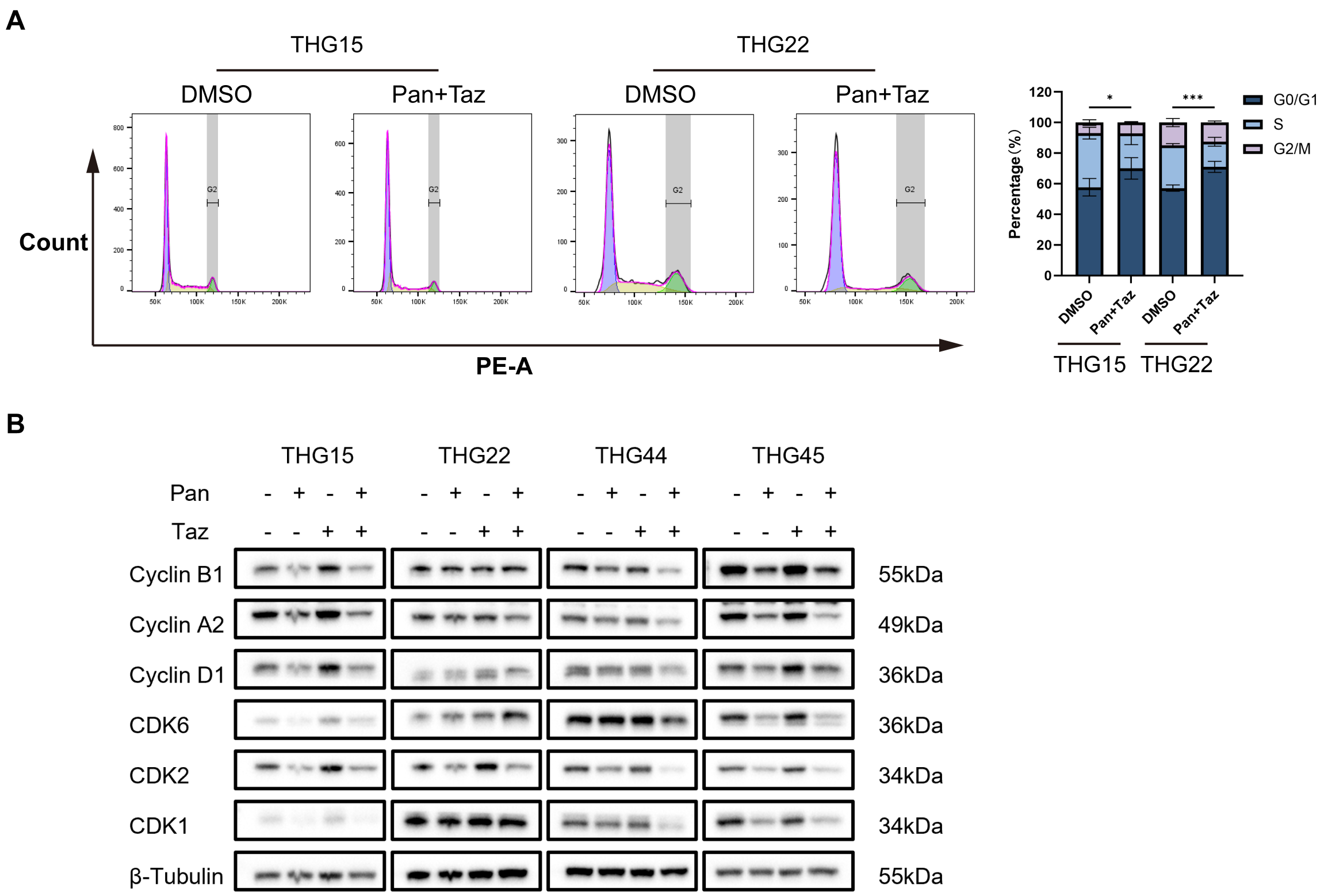

Figure S7 Time-dependent cell cycle arrest in DMG GSCs following dual-agent treatment. (A) Flow cytometric analysis of cell cycle distribution in THG15 and THG22 GSCs after 5 days of dual-agent treatment. Proportions of cells in each cell-cycle phase are quantified on the right, with statistical significance indicated for the S phase fraction. (B) Immunoblot analysis of cell cycle regulatory proteins, with β-tubulin serving as a loading control, in four DMG GSC cultures after 5 days of drug treatment. Pan, panobinostat; Taz, tazemetostat; *, $P<0.05$; **, $P<0.01$; ***, $P<0.001$.

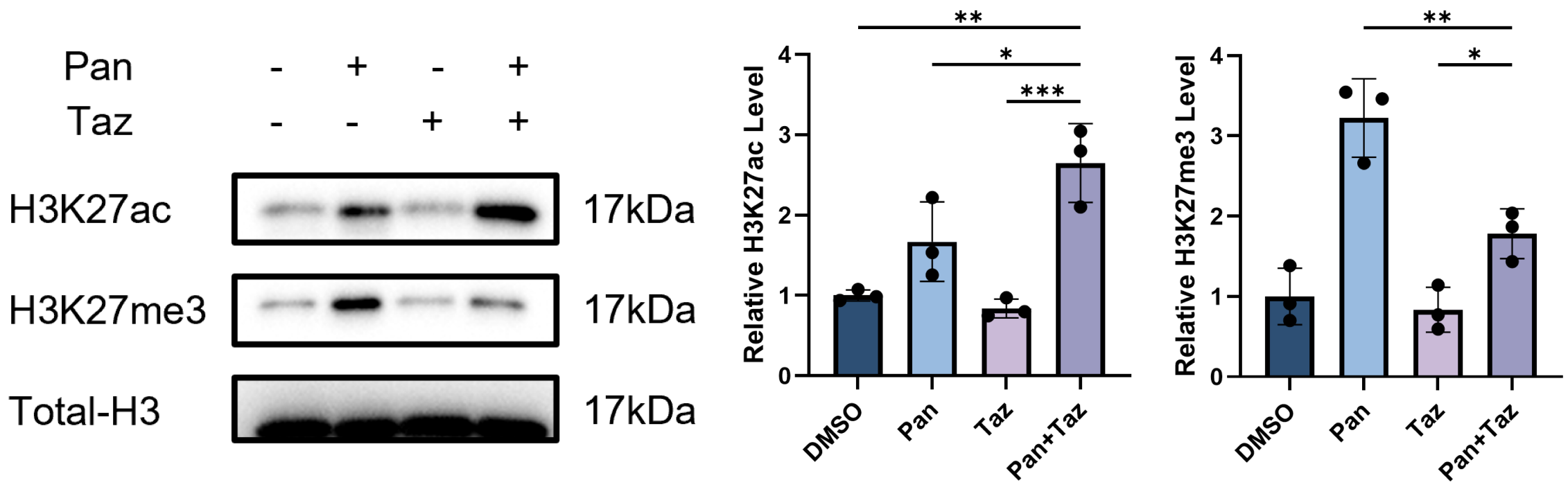

Figure S8 Alterations in epigenetic histone modifications in THG15 GSCs following dual-agent treatment. Immunoblot analysis of global H3K27ac and H3K27me3 levels in DMG GSCs following drug treatment. Quantification of relative signal intensity is shown on the right. This experiment was performed as an independent validation in the present study. Pan, panobinostat; Taz, tazemetostat; *, *P*<0.05; **, *P*<0.01; ***, *P*<0.001.
